# Supplementary figures and images for: Evidence for key individual characteristics associated with outcomes following combined first-line interventions for knee osteoarthritis: A systematic review
Source: PLoS One. 2023 Apr 11;18(4):e0284249. doi: 10.1371/journal.pone.0284249 (PMC10089365; doi:10.1371/journal.pone.0284249)

**Follow up duration in months**


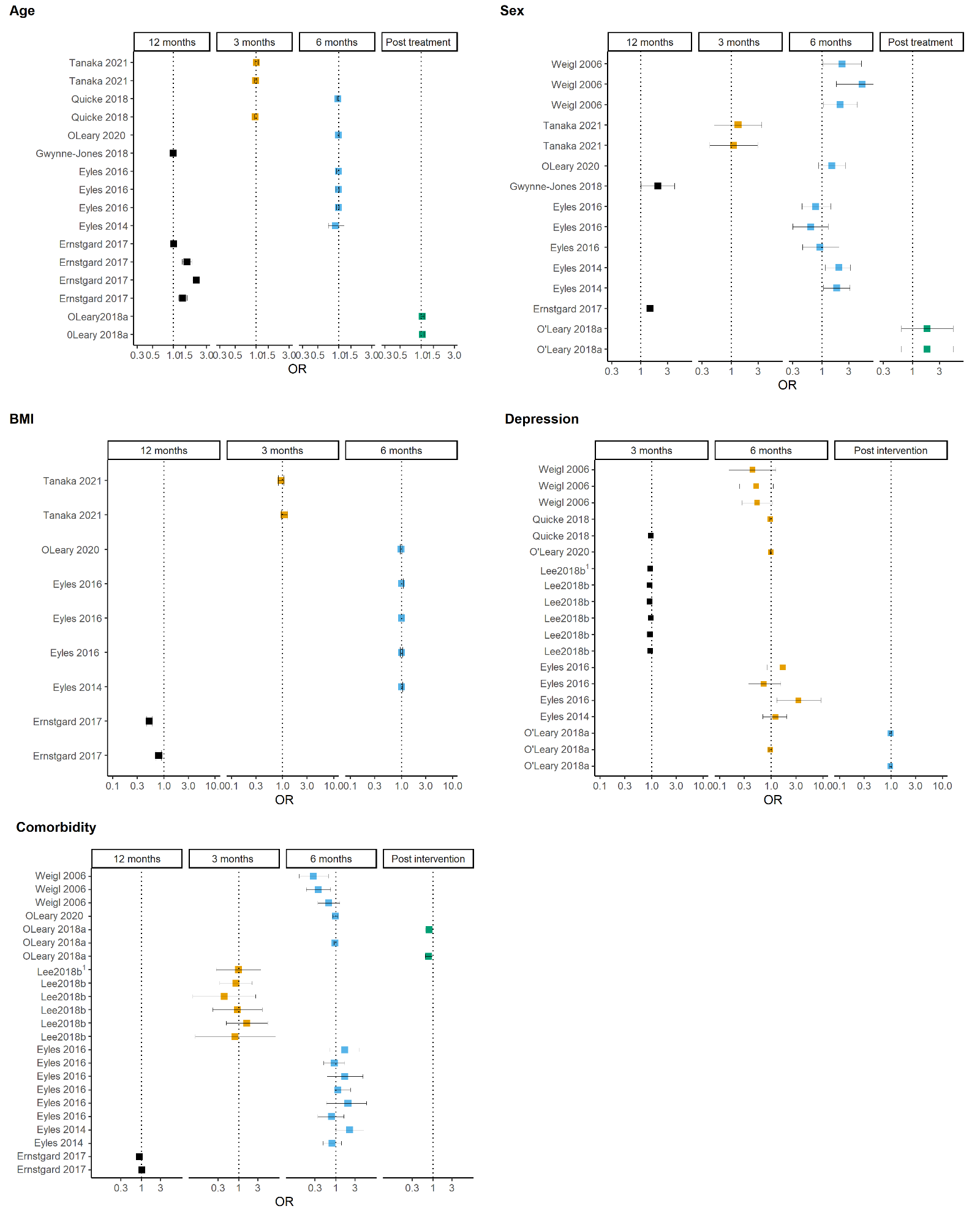


**Joint measured**


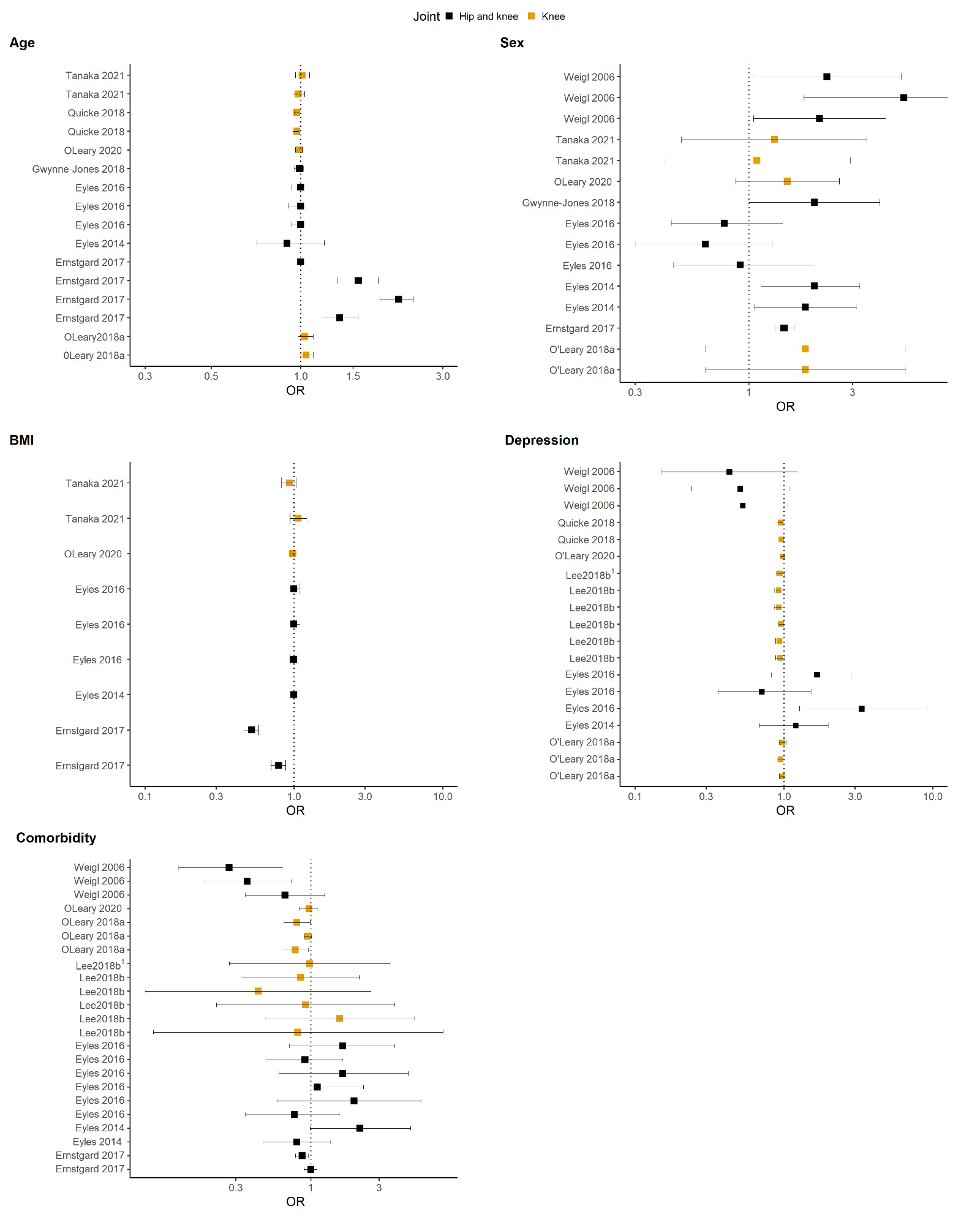

Supplement: S1 Fig — (DOCX) [file pone.0284249.s001.docx]
