# Supplementary material for: Evidence for key individual characteristics associated with outcomes following combined first-line interventions for knee osteoarthritis: A systematic review
Source: PLoS One. 2023 Apr 11;18(4):e0284249. doi: 10.1371/journal.pone.0284249 (PMC10089365; doi:10.1371/journal.pone.0284249)
Supplement: S2 Table — (DOCX) [file pone.0284249.s003.docx]

## **Search Strategies by Individual Databases**

“knee osteoarth*” OR “knee OA” OR “knee arthritis”

AND

Exercis* OR Strength* OR Balance OR Neuromuscular OR Education OR “self management” OR  “Weight loss” OR “weight management” OR intervention OR  “First line”  OR   multidisciplinary OR “multi disciplinary” OR nonsurgical OR “non surgical” OR nonpharmacological OR “non pharmacological” OR “core treatment” OR “GLAD” OR “Good Life Arthritis Denmark” OR “Better Management Osteoarthritis” OR “multicomponent” OR “multi component” OR nonoperative OR “non operative” OR “multi modal” OR multimodal

AND

Assoc* OR predict* OR correlat* OR “baseline characteristics” OR prognos* OR response* OR outcome* OR relationship* OR relate* OR factor*

| Database: MEDLINE (via EBSCO) | | | |
| --- | --- | --- | --- |
| Date of initial search: From inception to 4/06/2021  Date of most recent search: 4/6/2021 to 19/10/2022 | | | |
| Symbols used in this document:   1. “ “ finds a phrase . 2. Asterisk (*) finds various endings to a word stem 3. Limiters “human" | | | |
| **Search #** | **Concept** | **Search Terms/Strategy** | **# of Results** |
| # 1 | Knee osteoarthritis | TI (“knee osteoarth*” OR “knee OA” OR “knee arthritis” ) OR AB ( “knee osteoarth*” OR “knee OA” OR “knee arthritis” ) | 14 504 (initial search)  2 737 (June 2021 – Oct 2022) |
| # 2 | Multimodal nonsurgical and non-pharmacological intervention  Title OR Abstract TI OR AB | TI (Exercis* OR Strength* OR Balance OR Neuromuscular OR Education OR “self management” OR  “Weight loss” OR “weight management” OR intervention OR  “First line”  OR   multidisciplinary OR “multi disciplinary” OR nonsurgical OR “non surgical” OR nonpharmacological OR “non pharmacological” OR “core treatment” OR “GLAD” OR “Good Life Arthritis Denmark” OR “Better Management Osteoarthritis” OR “multicomponent” OR “multi component” OR nonoperative OR “non operative” OR “multi modal”) OR AB (Exercis*…….. | 2 539 704 (initial search)  346 289 (June 2021 – Oct 2022) |
| # 3 | Prediction Methods Association or Factors  (Title or abstract search) | TI (Assoc* OR predict* OR correlat* OR “baseline characteristics” OR prognos* OR response* OR outcome* OR relationship* OR relate* OR factor*) OR AB (Assoc* OR predict* OR correlat*……) | 12 798 4118 (initial search)  1 353 517 (June 2021 – Oct 2022) |
| # 4 |  | #1 AND #2 AND # 3 | 3332 (initial search)  707 (June 2021 to Oct 2022) |

| Database: CINAHL (via EBSCO) | | | |
| --- | --- | --- | --- |
| Date of initial search: From inception to 4/06/2021  Date of most recent search: 4/6/2021 to 19/10/2022 | | | |
| Symbols used in this document:   1. “ “ finds a phrase . 2. Asterisk (*) finds various endings to a word stem | | | |
| **Search #** | **Concept** | **Search Terms/Strategy** | **# of Results** |
| # 1 | Knee osteoarthritis | TI (“knee osteoarth*” OR “knee OA” OR “knee arthritis”) OR AB (“knee osteoarth*” OR “knee OA” OR “knee arthritis”) | 8787 (initial search)  1335 (June 2021 to Oct 2022) |
| # 2 | Multimodal nonsurgical and non-pharmacological intervention  Title OR Abstract TI OR AB | TI (Exercis* OR Strength* OR Balance OR Neuromuscular OR Education OR “self management” OR  “Weight loss” OR “weight management” OR intervention OR  “First line”  OR   multidisciplinary OR “multi disciplinary” OR nonsurgical OR “non surgical” OR nonpharmacological OR “non pharmacological” OR “core treatment” OR “GLAD” OR “Good Life Arthritis Denmark” OR “Better Management Osteoarthritis” OR “multicomponent” OR “multi component” OR nonoperative OR “non operative” OR “multi modal”) OR AB (Exercis*…….. | 1002 031 (initial search)  116 281 (June 2021 to Oct 2022) |
| # 3 | Prediction Methods Association or Factors  Title OR Abstract  TI or AB | TI (Assoc* OR predict* OR correlat* OR “baseline characteristics” OR prognos* OR response* OR outcome* OR relationship* OR relate* OR factor*) OR AB (Assoc* OR predict* OR correlat….) | 2 629 458 (initial search)  313 406 (June 2021 to Oct 2022) |
| # 4 |  | #1 AND #2 AND # 3 | 2 085 (initial search)  344 (June 2021 to Oct 2022) |
|  | | | |
| Database: SCOPUS | | | |
| Date of initial search: From inception to 4/06/2021  Date of most recent search: 4/6/2021 to 19/10/2022 | | | |
| Symbols used in this document:   1. “ “ finds a phrase . 2. Asterisk (*) finds various endings to a word stem | | | |
| **Search #** | **Concept** | **Search Terms/Strategy** | **# of Results** |
| # 1 | Knee osteoarthritis | TI (“knee osteoarth*” OR “knee OA” OR “knee arthritis” ) OR AB ( “knee osteoarth*” OR “knee OA” OR “knee arthritis” ) | 16 798 |
| # 2 | Multimodal nonsurgical and non-pharmacological intervention  Title OR Abstract TI OR AB | TI (Exercis* OR Strength* OR Balance OR Neuromuscular OR Education OR “self management” OR  “Weight loss” OR “weight management” OR intervention OR  “First line”  OR   multidisciplinary OR “multi disciplinary” OR nonsurgical OR “non surgical” OR nonpharmacological OR “non pharmacological” OR “core treatment” OR “GLAD” OR “Good Life Arthritis Denmark” OR “Better Management Osteoarthritis” OR “multicomponent” OR “multi component” OR nonoperative OR “non operative” OR “multi modal”) OR AB (Exercis*…….. | 6 079 336 |
| # 3 | Prediction Methods Association or Factors  Title or Abstract | TI (Assoc* OR predict* OR correlat* OR “baseline characteristics” OR prognos* OR response* OR outcome* OR relationship* OR relate* OR factor*) OR AB (Assoc* …..) | 26 769 606 |
| # 4 |  | #1 AND #2 AND # 3 | 3796 (initial search)  983 (June 2021 to Oct 2022) |

| Database: WEB OF SCIENCE CORE COLLECTION | | | |
| --- | --- | --- | --- |
| Date of initial search: From inception to 4/06/2021  Date of most recent search: 4/6/2021 to 19/10/2022 | | | |
| Symbols used in this document:   1. ““finds a phrase. 2. Asterisk (*) finds various endings to a word stem | | | |
| **Search #** | **Concept** | **Search Terms/Strategy** | **# of Results** |
| # 1 | Knee osteoarthritis | TI (“knee osteoarth*” OR “knee OA” OR “knee arthritis” ) OR AB ( “knee osteoarth*” OR “knee OA” OR “knee arthritis” ) | 17 970 (initial search)  2657 (June 2021 to Oct 2022) |
| # 2 | Multimodal nonsurgical and non-pharmacological intervention  Title OR Abstract TI OR AB | TI (Exercis* OR Strength* OR Balance OR Neuromuscular OR Education OR “self management” OR  “Weight loss” OR “weight management” OR intervention OR  “First line”  OR   multidisciplinary OR “multi disciplinary” OR nonsurgical OR “non surgical” OR nonpharmacological OR “non pharmacological” OR “core treatment” OR “GLAD” OR “Good Life Arthritis Denmark” OR “Better Management Osteoarthritis” OR “multicomponent” OR “multi component” OR nonoperative OR “non operative” OR “multi modal”) OR AB (Exercis*…….. | 4 601 297 (initial search)  523 610  (June 2021 to Oct 2022) |
| # 3 | Prediction Methods Association or Factors | Assoc* OR predict* OR correlat* OR “baseline characteristics” OR prognos* OR response* OR outcome* OR relationship* OR relate* OR factor* | 23 585 994 (initial search)  1 866 001 (June 2021 to Oct 2022) |
| # 4 |  | #1 AND #2 AND # 3 | 3621 (initial search)  620 (June 2021 to Oct 2022) |

| Database: Cochrane Library (Trials) | | | |
| --- | --- | --- | --- |
| Date of initial search: From inception to 4/06/2021  Date of most recent search: 4/6/2021 to 19/10/2022 | | | |
| Symbols used in this document:   1. “ “ finds a phrase . 2. Asterisk (*) finds various endings to a word stem | | | |
| **Search #** | **Concept** | **Search Terms/Strategy** | **# of Results** |
| **# 1** | Single line approach  Title abstract and keyword for all 3 lines | “knee osteoarth*” OR “knee OA” OR “knee arthritis”  AND  Exercis* OR Strength* OR Balance OR Neuromuscular OR Education OR “self management” OR  “Weight loss” OR “weight management” OR intervention OR  “First line”  OR   multidisciplinary OR “multi disciplinary” OR nonsurgical OR “non surgical” OR nonpharmacological OR “non pharmacological” OR “core treatment” OR “GLAD” OR “Good Life Arthritis Denmark” OR “Better Management Osteoarthritis” OR “multicomponent” OR “multi component” OR nonoperative OR “non operative” OR “multi modal” OR multimodal  AND  Assoc* OR predict* OR correlat* OR “baseline characteristics” OR prognos* OR response* OR outcome* OR relationship* OR relate* OR factor* | 1489 (initial search to June 2021)  290 (June 2021 to October 2022) |
