# Supplementary material for: Evidence for key individual characteristics associated with outcomes following combined first-line interventions for knee osteoarthritis: A systematic review
Source: PLoS One. 2023 Apr 11;18(4):e0284249. doi: 10.1371/journal.pone.0284249 (PMC10089365; doi:10.1371/journal.pone.0284249)
Supplement: S3 Table — (DOCX) [file pone.0284249.s004.docx]

Risk of bias assessment using Quality in Prognostic Studies (QUIPS) for the 32 studies included in the systematic review

| Study | Study Participation | Study Attrition | Prognostic Factor Measurement | Outcome assessment | Adjustment for other prognostic factors | Statistical Analysis and Reporting | Overall Risk of Bias |
| --- | --- | --- | --- | --- | --- | --- | --- |
| Primary objective: Studies examining baseline participant characteristics associated with improvements in pain and function | | | | | | | |
| Pihl 2021 | Moderate | Low | Low | Low | Low | Low | Low |
| Unevik 2020 | Low | High | Low | Moderate | Low | Moderate | Moderate |
| Lawford 2021 | Low | Low | Moderate | Moderate | Low | Low | Low |
| O'Leary 2018a | Moderate | Moderate | Low | Low | Moderate | Moderate | Moderate |
| O'Leary 2020 | Low | Low | Low | Moderate | Moderate | Moderate | Moderate |
| Legha 2020 | Low | Low | Moderate | Low | Moderate | Low | Moderate |
| Degerstedt 2020 | Low | Low | Low | Low | Low | Low | Low |
| Dell'Isola 2020 | Moderate | High | Moderate | Low | Low | Low | Moderate |
| Ernstgard 2017 | Low | Moderate | Moderate | Moderate | Moderate | Low | Moderate |
| Gwynne-Jones 2018 | Moderate | Moderate | Moderate | Low | Low | Low | Moderate |
| Eyles 2014 | Moderate | Moderate | Moderate | Low | Moderate | Low | Moderate |
| Eyles 2016 | Moderate | Moderate | Low | Low | Moderate | Moderate | Moderate |
| Lee 2018a | Low | Moderate | Low | Moderate | High | High | Moderate |
| O'Leary 2018b | Moderate | Moderate | Low | Low | Low | Low | Moderate |
| Tanaka 2021 | Low | High | Low | Low | Moderate | Moderate | Moderate |
| Weigl 2006 | Low | Moderate | Moderate | Moderate | Moderate | Moderate | Moderate |
| Quicke 2018 | Low | Low | Low | Low | Low | Moderate | Low |
| Chang 2019 | Low | Moderate | Low | Low | Low | Low | Low |
| Knoop 2014 | Low | Low | Moderate | Low | Low | Low | Low |
| Hall 2017 | Low | Moderate | Low | Low | Low | Low | Low |
| Lee 2018b | Low | Low | Low | Low | Low | Low | Low |
| Lee 2017 | Moderate | Moderate | Moderate | Low | Moderate | High | Moderate |
| Baumbach 2021 | Low | Low | Low | Low | N/A | Low | Low |
| Lawford 2018 | Low | Low | Low | Low | Moderate | Low | Low |
| Skou 2018 | Low | Moderate | Moderate | Low | Low | Low | Low |
| Peat 2022 | Moderate | Moderate | Moderate | Low | Moderate | Moderate | Moderate |
| Nelligan 2022 | Low | Low | Low | Low | Moderate | Moderate | Low |
| Henrikson 2022 | Low | Low | Moderate | Low | Moderate | N/A | Low |
|  |  |  |  |  |  |  |  |
| Secondary objective: Studies examining baseline patient characteristics associated with change in willingness to undertake surgery (or undertake knee joint replacement) | | | | | | | |
| Teoh 2017 | Moderate | Moderate | Low | Moderate | Moderate | Moderate | Moderate |
| Dell’lsola 2021 | Moderate | Moderate | Moderate | Moderate | Low | Moderate | Moderate |
| Gwynne-Jones 2020 | Low | Low | Moderate | Low | Moderate | Low | Moderate |
| Gustafsson 2022 | Low | Moderate | Moderate | Low | Moderate | Low | Moderate |
