# Supplementary material for: Evidence for key individual characteristics associated with outcomes following combined first-line interventions for knee osteoarthritis: A systematic review
Source: PLoS One. 2023 Apr 11;18(4):e0284249. doi: 10.1371/journal.pone.0284249 (PMC10089365; doi:10.1371/journal.pone.0284249)
Supplement: S4 Table — (DOCX) [file pone.0284249.s005.docx]

**Effect measures and individual study results for prognostic factors of age, sex, BMI, depression, comorbidities, and baseline imaging**

**Primary outcome: change in pain or function from baseline to follow up**

| 1. **Prognostic factor: Baseline imaging** | | | | | | |
| --- | --- | --- | --- | --- | --- | --- |
| **Study** | **Outcome Measure** | **Imaging Measure** | **Effect measure** | **Imaging Reference** | **Results** | **Conclusions** |
| Knoop  2014 | WOMAC physical function  NRS pain (0-10)  Responder criteria using OMERACT/OARSI | MRI (cartilage integrity, BMLs, osteophyte formation, effusion/synovitis, and meniscal abnormalities | OR  ß coefficient | Dichotomised  KL Grade 2/3 (vs Grade 0/1) for PF and TF joint | Evidence higher severity in PF joint (Grade 2/3) associated with less improvement in WOMAC function (adjusted ß= -5.0 (95%CI  -8.8, 1.2) and less improvement NRS pain and lower odds treatment response (OR remained > 1 indicating positive response) | All grades of OA severity on MRI are associated with a positive response; however, the response may be reduced with advanced PF OA |
| O’Leary 2020 | Global rating of change (GRoC)  15-point Likert scale  Responder (+2 to +7) Nonresponder (-7 to +1) | Radiographic, CT or MRI reported in medical notes (absent, mild, moderate, or severe) | OR | Mild (vs absent)  Moderate (vs absent)  Severe (vs absent)  Reported in medial, lateral and PF compartments | Medial compartment was included in the final multivariable model (based on univariable screening)  Adjusted OR for severe medial knee OA vs mild= 3.11 (95%CI 1.04,9.3)  OR for moderate medial knee OA vs mild= 2.39 (95% CI 0.84, 6.79) | Authors concluded that severe medial compartment changes on imaging were associated with poorer response compared to mild changes |
| Lee  2018b‡ | Pain and functional trajectories (WOMAC measured weekly over 12 weeks) | Radiographical KL grade | OR | KL grade 3-4 (vs Grade 0-2) | KL grades are evenly distributed among the four trajectory groups.  Authors reported no significant association between KL grade and trajectory groups  OR was positive for KL grade 3-4 (vs 0-2) for all trajectory groups  E.g., OR higher pain, delayed improvement KL grade 3-4 (v 0-2) = 1.67 (95%CI 0.52,5.37). | Higher KL grade (3-4) had an increased odds of being in each pain trajectory category (OR all > 1)  Similar results were found for functional trajectories except for lower function, gradual improvement OR=0.92 (0.32,3.63) |
| Henriksen 2022† | Change in KOOS pain subscale at 9 weeks | Radiographical KL grade |  | KL grade 2-3 (v KL grade 4) | This study was an effect modifier analysis comparing GLAD to saline injections.  Mean difference was presented between subgroups for both interventions. However, when comparing mean KOOS change between KL grade 2-3 and KL grade 4 for the GLAD intervention, the difference was minimal  KL grade 2-3 mean KOOS change was 9.5 (1.6) and for KL grade 4 was 7.0 (2.0) | Evidence for no real difference in mean change KOOS pain score between KL grade 2-3 and KL grade 4  Small sample in GLAD intervention group (n=102) and no association measures were reported as the main aim of the study was to compare both interventions |
|  |  |  |  |  |  |  |
| ‡Lee 2018b reported multiple outcome variables. 4 trajectories of improvement were identified (1) Lower pain/ early improvement (2) Moderate pain, early improvement (3) Higher pain, delayed improvement (4) Higher pain, no improvement. OR > 1 favours KL grade 3-4 (compared to KL 2-3) being in this trajectory group.  †Henriksen 2022 was a reanalysis of RCT looking at treatment effect modifiers. The main aim of this study was to examine factors that modify the treatment effect of GLAD on pain compared to that of intra-articular saline injections. The mean difference was an unadjusted effect estimate. | | | | | | |

1. **Prognostic Factor: Age**

| **Study** | **Effect measure**  ***= un-adjusted** | **Outcome measure** | **Follow up duration(months)** | **Outcome reference (Original study)** | **Age reference** | **Effect size (Original study)** | **Lower 95 % CI** | **Upper 95 % CI** | **Outcome reference**  **(rescaled)** | **Age Reference (rescaled)** | **Effect Size (rescaled)** | **Lower 95 %**  **CI** | **Higher 95 % CI** |
| --- | --- | --- | --- | --- | --- | --- | --- | --- | --- | --- | --- | --- | --- |
| OLeary 2020 | OR * | GROC | 6 | Non-responder =1 | Per one year | 1.01 | 0.99 | 1.04 | Responder =1 | Per one year | 0.99 | 0.96 | 1.01 |
| Eyles 2016 | OR * | WOMAC-G | 6 | Non-responder =1 | Per one year | 1 | 0.99 | 1.08 | Responder =1 | Per one year | 1 | 0.93 | 1.01 |
| Eyles 2016 | OR * | Transition scale | 6 | Non-responder =1 | Per one year | 1 | 0.99 | 1.1 | Responder =1 | Per one year | 1 | 0.91 | 1.01 |
| Eyles 2016 | OR * | WOMAC-G + transition | 6 | Non-responder =1 | Per one year | 1 | 1 | 1.07 | Responder =1 | Per one year | 1 | 0.93 | 1 |
| Gwynne-Jones 2018 | OR | OKS | 12 | Non-responder =1 | Per one year^[[1]](#footnote-1)^ | 1.0554 | 1.016 | 1.094 | Responder =1 | Per one year | 0.95 | 0.91 | 0.98 |
| 0Leary 2018a | OR | OMERACT-OARSI | Post treatment | Non-responder =1 | Per one year | 0.97 | 0.91 | 1.02 | Responder =1 | Per one year | 1.03 | 0.98 | 1.1 |
| 0Leary 2018a | OR | WOMAC pain | Post treatment | Non-responder =1 | Per one year | 0.96 | 0.91 | 1.02 | Responder =1 | Per one year | 1.04 | 0.98 | 1.1 |
| Ernstgard 2017 | OR | Self-report PA minimum | 3 and 12 | Non-responder =1 | 55-64 yrs. (v 22-54) | 0.74 | 0.64 | 0.85 | Responder =1 | 55-64 yrs. (v 22-54) | 1.35 | 1.18 | 1.56 |
| Ernstgard 2017 | OR | Self-report PA minimum | 3 and 12 | Non-responder =1 | 65-74 yrs. (v 22-54) | 0.47 | 0.42 | 0.54 | Responder =1 | 65-74 yrs. (v 22-54) | 2.13 | 1.85 | 2.38 |
| Ernstgard 2017 | OR | Self-report PA minimum | 3 and 12 | Non-responder =1 | 75-100 yrs. (v 22-54) | 0.64 | 0.55 | 0.75 | Responder =1 | 75-100 yrs. (v 22-54) | 1.56 | 1.33 | 1.82 |
| Ernstgard 2017 | OR | PA minimum | 3 and 12 |  |  |  |  |  | Responder =1 | 22-54 yrs. | 1 |  |  |
| Eyles 2014 | OR * | WOMAC-G | 6 | Responder =1 | Per one year | 0.9 | 0.71 | 1.2 | Responder =1 | Per one year | 0.9 | 0.7 | 1.2 |
| Quicke 2018 | OR | OMERACT-OARSI | 3 | Responder =1 | Per one year | 0.97 | 0.94 | 0.99 | Responder =1 | Per one year | 0.97 | 0.94 | 0.99 |
| Quicke 2018 | OR | OMERACT-OARSI | 6 | Responder =1 | Per one year | 0.97 | 0.95 | 0.99 | Responder =1 | Per one year | 0.97 | 0.95 | 0.99 |
| Gwynne-Jones 2018 | OR | OKS | 12 | Responder =1 | Per one year^[[2]](#footnote-2)^ | 0.9878 | 0.9515 | 1.0247 | Responder =1 | Per one year | 0.99 | 0.95 | 1.02 |
| Tanaka 2021 | OR | OKS | 3 | Responder =1 | Per one year | 0.983 | 0.937 | 1.031 | Responder =1 | Per one year | 0.98 | 0.94 | 1.03 |
| Tanaka 2021 | OR | NRS | 3 | Responder =1 | Per one year | 1.014 | 0.964 | 1.068 | Responder =1 | Per one year | 1.01 | 0.96 | 1.07 |
| Gwynne-Jones 2018 | MD | OKS | 12 | Negative= deterioration on OKS | Age per 5 years (v previous 5 years) | -1 | -1.6 | -0.5 |  |  |  |  |  |
| Legha 2020 | MD | WOMAC pain | 6 | Positive= less improvement pain | per one year | 0.06 | 0.04 | 0.09 |  |  |  |  |  |
| Legha 2020 | MD | WOMAC function | 6 | Positive= less improvement in pain | per one year | 0.2 | 0.11 | 0.29 |  |  |  |  |  |
| Henrikson 2022† | MD * | KOOS pain | 9 weeks | Positive= improvement in KOOS pain | Dichotomised (above/ below the median age | Mean KOOS for GLAD only:  Above median age = 6.8 (1.7 SE)  Below median age= 10.8 (2 SE.) | | |  | Above median age (v below median age) | MD= 4 | -1.2 | 9.2 |
| Lee 2018a | HR * | WOMAC pain | Up to 3 months | Responder =1 | per one year | 1.01 | 0.99 | 1.03 |  |  |  |  |  |
| Lee 2018a | HR * | WOMAC function | Up to 3 months | Responder =1 | per one year | 1.02 | 1 | 1.04 |  |  |  |  |  |
| Quicke 2018 | Beta coefficient (ß) | WOMAC pain | 3 | positive ß= larger increase pain at follow up | per one year | 0.06 | 0.03 | 0.08 |  |  |  |  |  |
| Quicke 2018 | Beta coefficient (ß) | WOMAC function | 3 | positive ß= larger reduction function | per one year | 0.15 | 0.05 | 0.21 |  |  |  |  |  |
| Quicke 2018 | Beta coefficient (ß) | WOMAC pain | 6 | positive ß= larger increase pain | per one year | 0.05 | 0.02 | 0.08 |  |  |  |  |  |
| Quicke 2018 | Beta coefficient (ß) | WOMAC function | 6 | positive ß= larger reduction function | per one year | 0.19 | 0.09 | 0.29 |  |  |  |  |  |
| Dell’lsola 2020 | Beta coefficient (ß) | NRS | 3 | positive ß= larger increase pain | per one year | 0 | 0 | 0.01 |  |  |  |  |  |
| Dell’lsola 2020 | Beta coefficient (ß) | NRS | 12 | positive ß= larger increase pain  Negative ß= reduction in pain | per one year | 0.01 | 0.01 | 0.02 |  |  |  |  |  |

†Henriksen 2022 was a reanalysis of RCT looking at treatment effect modifiers. They planned to look at association measures at baseline, but this was not performed. The main aim of this study was to examine factors that modify the treatment effect of GLAD on pain compared to that of intra-articular saline injections.

1. **Prognostic Factor: Sex**

| **Study** | **Effect measure**  ***Unadjusted** | **Outcome measure** | **Follow up duration (months)** | **Outcome reference (Original study)** | **Sex Target group (Original study)** | **Effect size (Original study)** | **Lower 95 % CI** | **Higher 95 % CI** | **Outcome Reference**  **(rescaled)** | **Sex Target**  **(Rescaled female)** | **Effect Size (female responder)** | **Lower 95 % CI** | **Higher 95 % CI** |
| --- | --- | --- | --- | --- | --- | --- | --- | --- | --- | --- | --- | --- | --- |
| Weigl 2006 | OR | WOMAC-G | 6 | Responder= 1 | Female=1 | 2.11 | 1.05 | 4.25 | Responder=1 | Female=1 | 2.11 | 1.05 | 4.25 |
| Weigl 2006 | OR | Transition scale | 6 | Responder= 1 | Female=1 | 2.28 | 1.04 | 5 | Responder=1 | Female=1 | 2.28 | 1.04 | 5 |
| Weigl 2006 | OR | WOMAC-G + transition | 6 | Responder= 1 | Female=1 | 5.16 | 1.79 | 14.84 | Responder=1 | Female=1 | 5.16 | 1.79 | 14.84 |
| Eyles 2014 | OR * | WOMAC-G | 6 | Responder= 1 | Male=1 | 0.5 | 0.31 | 0.88 | Responder=1 | Female=1 | 2 | 1.14 | 3.23 |
| Eyles 2014 | OR * | WOMAC-G | 6 | Responder= 1 | Male=1 | 0.55 | 0.32 | 0.94 | Responder=1 | Female=1 | 1.82 | 1.06 | 3.13 |
| Gwynne-Jones 2018 | OR | OKS | 12 | Responder= 1 | Male=1 | 0.5 | 0.25 | 1 | Responder=1 | Female=1 | 2 | 1 | 4 |
| Eyles 2016 | OR * | WOMAC-G | 6 | Non-responder = 1 | Female=1 | 1.1 | 0.5 | 2.23 | Responder=1 | Female=1 | 0.91 | 0.45 | 2 |
| Eyles 2016 | OR * | Transition scale | 6 | Non-responder = 1 | Female=1 | 1.6 | 0.78 | 3.29 | Responder=1 | Female=1 | 0.63 | 0.3 | 1.28 |
| Eyles 2016 | OR * | WOMAC-G + transition | 6 | Non-responder = 1 | Female=1 | 1.3 | 0.7 | 2.28 | Responder=1 | Female=1 | 0.77 | 0.44 | 1.43 |
| 0Leary 2018a | OR | OMERACT-OARSI | Post-treatment | Non-responder = 1 | Female=1 | 0.55 | 0.19 | 1.59 | Responder=1 | Female=1 | 1.82 | 0.63 | 5.26 |
| 0Leary 2018a | OR | WOMAC pain | Post-treatment | Non-responder = 1 | Female=1 | 0.55 | 0.19 | 1.6 | Responder=1 | Female=1 | 1.82 | 0.63 | 5.26 |
| Ernstgard 2017 | OR | Self-report PA minimum | 12 | Non-responder = 1 | Female=1 | 0.69 | 0.62 | 0.75 | Responder=1 | Female=1 | 1.45 | 1.33 | 1.61 |
| OLeary 2020 | OR * | GROC | 6 | Non-responder = 1 | Male=1 | 1.5 | 0.87 | 2.6 | Responder=1 | Female=1 | 1.5 | 0.87 | 2.6 |
| Tanaka 2021 | OR | OKS | 3 | Responder= 1 | Male=1 | 0.765 | 0.288 | 2.029 | Responder=1 | Female=1 | 1.31 | 0.49 | 3.47 |
| Tanaka 2021 | OR | NRS | 3 | Responder= 1 | Male=1 | 0.914 | 0.341 | 2.449 | Responder=1 | Female=1 | 1.09 | 0.41 | 2.93 |
| Lee 2018a | HR * | WOMAC pain | Up to 12 weeks | Responder= 1 | Female=1 | 1.32 | 0.87 | 2.01 |  |  |  |  |  |
| Lee 2018a | HR * | WOMAC function | Up to 12 weeks | Responder= 1 | Female=1 | 1.29 | 0.85 | 1.96 |  |  |  |  |  |
| Legha 2020 | MD | WOMAC pain | 6 | Positive= less improvement in pain (v reference) | Female=1 | -0.32 | -0.79 | 0.16 |  |  |  |  |  |
| Legha 2020 | MD | WOMAC function | 6 | Positive= less improvement in pain (v reference) | Female=1 | -0.75 | -2.32 | 0.83 |  |  |  |  |  |
| Gwynne-Jones 2018 | MD | OKS | 12 | Negative= deterioration OKS | Male=1 | -0.7 | -2.8 | 1.4 |  |  |  |  |  |
| Henrikson 2022† | MD * | KOOS pain | 9 weeks | Positive= improvement in KOOS pain | Male and Female | Mean KOOS for GLAD only:  Male = 9.2 (1.7 SE)  Female = 7.6 (1.9 SE) | | |  | Male (v female) | MD= 1.6 | -3.6 | 6.8 |

†Henriksen 2022 was a reanalysis of RCT looking at treatment effect modifiers. They planned to look at association measures at baseline, but this was not performed. The main aim of this study was to examine factors that modify the treatment effect of GLAD on pain compared to that of intra-articular saline injections.

1. **Prognostic Factor: BMI (Body Mass Index)**

| **Study** | **Effect**  **Measure *unadjusted** | **Outcome Measure** | **Follow up duration (months)** | **Outcome Reference (Original study)** | **BMI target group (Original study)** | **Effect size (Original study)** | **Lower 95 % CI** | **Higher 95 % CI** | **Outcome**  **Reference (rescaled)** | **BMI**  **Target group** | **Effect size (BMI)** | **Lower 95 % CI** | **Higher 95 % CI** |
| --- | --- | --- | --- | --- | --- | --- | --- | --- | --- | --- | --- | --- | --- |
| Eyles 2014 | OR * | WOMAC-G | 6 | Responder=1 | per k/m2 | 1 | 0.98 | 1.05 | Responder=1 | per k/m2 | 1 | 0.98 | 1.05 |
| OLeary 2020 | OR * | GROC | 6 | Non-responder =1 | per k/m2 | 1.02 | 0.98 | 1.05 | Responder=1 | per k/m2 | 0.98 | 0.95 | 1.02 |
| Ernstgard 2017 | OR | Self-report PA minimum | 12 | Non-responder =1 | overweight=1 normal=0 | 1.27 | 1.14 | 1.42 | Responder=1 | overweight=1  normal BMI=0 | 0.79 | 0.7 | 0.88 |
| Ernstgard 2017 | OR | Self-report PA minimum | 12 | Non-responder =1 | obese=1 normal BMI=0 | 1.93 | 1.71 | 2.17 | Responder=1 | obese=1  normal BMI=0 | 0.52 | 0.46 | 0.58 |
| Eyles 2016 | OR * | WOMAC-G | 6 | Non-responder =1 | per k/m2 | 1 | 0.95 | 1.06 | Responder=1 | per k/m2 | 1 | 0.94 | 1.05 |
| Eyles 2016 | OR * | Transition scale | 6 | Non-responder =1 | per k/m2 | 1 | 0.91 | 1.02 | Responder=1 | per k/m2 | 1 | 0.98 | 1.1 |
| Eyles 2016 | OR * | WOMAC-G + transition | 6 | Non-responder =1 | per k/m2 | 1 | 0.93 | 1.02 | Responder=1 | per k/m2 | 1 | 0.98 | 1.08 |
| Tanaka 2021 | OR | OKS | 3 | Responder=1 | per k/m2 | 1.069 | 0.936 | 1.22 | Responder=1 | per k/m2 | 1.07 | 0.94 | 1.22 |
| Tanaka 2021 | OR | NRS | 3 | Responder=1 | per k/m2 | 0.925 | 0.82 | 1.043 | Responder=1 | per k/m2 | 0.93 | 0.82 | 1.04 |
| Lee 2018a | HR * | WOMAC  pain | Up to 12 weeks | Responder=1 | per k/m2 | 0.98 | 0.96 | 1.01 |  |  |  |  |  |
| Lee 2018a | HR * | WOMAC  function | Up to 12 weeks | Responder=1 | per k/m2 | 0.98 | 0.95 | 1.00 |  |  |  |  |  |
| Legha 2020 | MD | WOMAC  pain | 6 | Positive= less improvement in pain (v reference group | overweight=1 normal BMI=0 | 0.06 | -0.55 | 0.68 |  |  |  |  |  |
| Legha 2020 | MD | WOMAC  pain | 6 | Positive= less improvement in pain (v reference group | obese=1 normal BMI=0 | 0.89 | 0.23 | 1.54 |  |  |  |  |  |
| Legha 2020 | MD | WOMAC  function | 6 | Positive= less improvement in pain (v reference group) | overweight=1 normal BMI=0 | -0.54 | -2.6 | 1.51 |  |  |  |  |  |
| Legha 2020 | MD | WOMAC  function | 6 | Positive= less improvement in pain (v reference group) | obese=1 normal BMI=0 | 2.34 | 0.12 | 4.56 |  |  |  |  |  |
| Henrikson 2022† | MD * | KOOS pain | 9 weeks | Positive= improvement in KOOS pain | Dichotomised (BMI > 30 and < 30) | Mean KOOS for GLAD only:  BMI > 30 = 3.4 (SE=2.6)  BMI < 30 = 10.1 (SE=1.5) | | |  | BMI < 30 (versus >30) | MD= 6.7 | 0.76 | 12.64 |
| Dell’lsola 2020 | Beta coefficient (ß) | NRS | 3 | positive ß= larger increase pain | per k/m2 | 0.02 | 0.02 | 0.03 |  |  |  |  |  |
| Dell’lsola 2020 | Beta coefficient (ß) | NRS | 12 | positive ß= larger increase pain | per k/m2 | 0.03 | 0.02 | 0.04 |  |  |  |  |  |

†Henriksen 2022 was a reanalysis of RCT looking at treatment effect modifiers. They planned to look at association measures at baseline, but this was not performed. The main aim of this study was to examine factors that modify the treatment effect of GLAD on pain compared to that of intra-articular saline injections.

1. **Prognostic Factor Depression**

| **Study** | **Effect Measure*unadjusted** | **Outcome Measure** | | **Follow up duration (months)** | **Outcome Reference**  **(Original study)** | **Depression**  **Target group (Original study)** | **Effect size (Original study)** | **Low 95 % CI** | **High**  **95 % CI** | **Outcome reference (rescaled)** | **Depression Target group (rescaled)** | **Effect Size**  **(rescaled)** | **Low**  **95 % CI** | **High 95 % CI** |
| --- | --- | --- | --- | --- | --- | --- | --- | --- | --- | --- | --- | --- | --- | --- |
| OLeary 2020 | OR * | | GROC | 6 | Non-responder=1 | per increase 1/42 DASS 21 | 1.02 | 0.99 | 1.05 | Responder=1 | per 1/42 DASS 21 increase | 0.98 | 0.95 | 1.01 |
| Eyles 2016 | OR | | WOMAC-G | 6 | Non-responder=1 | Yes (DASS ≥10) = 1 | 0.3 | 0.11 | 0.79 | Responder=1 | Yes (DASS ≥10) = 1 | 3.33 | 1.27 | 9.09 |
| Eyles 2016 | OR * | | Transition scale | 6 | Non-responder=1 | Yes (DASS ≥10) = 1 | 1.4 | 0.66 | 2.77 | Responder=1 | Yes (DASS ≥10) = 1 | 0.71 | 0.36 | 1.52 |
| Eyles 2016 | OR * | | WOMAC-G + transition | 6 | Non-responder=1 | Yes (DASS ≥10) = 1 | 0.6 | 0.35 | 1.22 | Responder=1 | Yes (DASS ≥10) = 1 | 1.67 | 0.82 | 2.86 |
| Eyles 2014 | OR * | | WOMAC-G | 6 | Responder=1 | Yes (DASS ≥14) = 1 | 1.2 | 0.68 | 1.98 | Responder=1 | Yes (DASS ≥14) = 1 | 1.2 | 0.68 | 1.98 |
| Weigl 2006 | OR | | WOMAC-G (18% improvement) | 6 | Responder=1 | No (HADS < 8) =1 | 1.87 | 0.95 | 3.67 | Responder=1 | Yes (HADS > 8) =1 | 0.53 | 0.27 | 1.05 |
| Weigl 2006 | OR | | Transition scale | 6 | Responder=1 | No (HADS < 8) =1 | 1.98 | 0.93 | 4.21 | Responder=1 | Yes (HADS > 8) =1 | 0.51 | 0.24 | 1.08 |
| Weigl 2006 | OR | | WOMAC-G + transition | 6 | Responder=1 | No (HADS < 8) =1 | 2.34 | 0.82 | 6.63 | Responder=1 | Yes (HADS > 8) =1 | 0.43 | 0.15 | 1.22 |
| Quicke 2018 | OR | | OMERACT-OARSI | 6 | Responder=1 | per increase 1/24 PHQ8 | 0.96 | 0.91 | 0.99 | Responder=1 | per increase 1/24 PHQ8 | 0.96 | 0.91 | 0.99 |
| Quicke 2018 | OR | | OMERACT-OARSI | 6 | Responder=1 | per increase 1/24 PHQ8 | 0.95 | 0.91 | 0.99 | Responder=1 | per increase 1/24 PHQ8 | 0.95 | 0.91 | 0.99 |
| 0Leary 2018a | OR | | OMERACT-OARSI | 3 | Non-responder=1 | Yes (> 16 CES-D) =1 | 1.03 | 0.98 | 1.08 | Responder=1 | Yes (> 16 CES-D) =1 | 0.97 | 0.93 | 1.02 |
| 0Leary 2018a | OR | | OMERACT-OARSI | 6 | Non-responder=1 | Yes (> 16 CES-D) =1 | 1.05 | 0.99 | 1.11 | Responder=1 | Yes (> 16 CES-D) =1 | 0.95 | 0.9 | 1.01 |
| 0Leary 2018a | OR | | WOMAC pain | 3 | Non-responder=1 | Yes (> 16 CES-D) =1 | 1.02 | 0.97 | 1.08 | Responder=1 | Yes (> 16 CES-D) =1 | 0.98 | 0.93 | 1.03 |
| Lee2018b† | OR | | WOMAC  pain | Trajectory-based 3 months | Higher pain no improvement (v lower pain, early improvement) | per increase 1/63 Beck-II | 1.06 | 1 | 1.13 | Responder=1 (lower pain, early improvement) | per increase 1/63 Beck-II | 0.94 | 0.88 | 1 |
| Lee2018b | OR | | WOMAC  pain | Trajectory-based 3 months | Higher pain, delayed improvement (v lower pain, early improvement) | per increase 1/63 Beck-II | 1.07 | 1.01 | 1.14 | Responder=1 | per increase 1/63 Beck-II | 0.93 | 0.88 | 0.99 |
| Lee2018b | OR | | WOMAC  pain | Trajectory-based 3 months | Moderate pain early improvement (v lower pain, early improvement) | per increase 1/63 Beck-II | 1.04 | 0.99 | 1.09 | Responder=1 | per increase 1/63 Beck-II | 0.96 | 0.92 | 1.01 |
| Lee2018b | OR | | WOMAC  Function | Trajectory-based 3 months | Lower function, delayed improvement (v higher function, early improvement) | per increase 1/63 Beck-II | 1.09 | 1.03 | 1.16 | Responder=1 (lower pain, early improvement) | per increase 1/63 Beck-II | 0.92 | 0.86 | 0.97 |
| Lee2018b | OR | | WOMAC  pain | Trajectory-based 3 months | Lower function, gradual improvement (v higher function, early improvement) | per increase 1/63 Beck-II | 1.09 | 1.02 | 1.16 | Responder=1 | per increase 1/63 Beck-II | 0.92 | 0.86 | 1 |
| Lee2018b | OR | | WOMAC  pain | Trajectory-based 3 months | Moderate function, early improvement (v higher function, early improvement) | per increase 1/63 Beck-II | 1.04 | 0.99 | 1.09 | Responder=1 | per increase 1/63 Beck-II | 0.94 | 0.89 | 1 |
| Legha 2020 | MD | | WOMAC  pain | 3 | Positive= less improvement in pain (v reference group) | Yes, anxious, or depressed=1 | 0.76 | 0.25 | 1.28 |  |  |  |  |  |
| Legha 2020 | MD | | WOMAC  function | 3 | Positive= less improvement in pain (v reference group | Yes, anxious, or depressed=1 | 1.93 | 0.18 | 3.68 |  |  |  |  |  |
| Pihl 2020 | MD | | 40metre walk test | 6 | Negative=less improvement | Yes depression=1 | 0.01 | -0.02 | 0.04 |  |  |  |  |  |
| Quicke 2018 | Beta coefficient (ß) | | WOMAC  pain | 6 | positive ß= larger increase pain | per 1/24 additional score | 0.11 | 0.05 | 0.17 |  |  |  |  |  |
| Quicke 2018 | Beta coefficient (ß) | | WOMAC  function | 3 | positive ß= larger reduction function | per 1/24 additional score | 0.23 | 0.03 | 0.42 |  |  |  |  |  |
| Quicke 2018 | Beta coefficient (ß) | | WOMAC  pain | 3 | positive ß= larger increase pain | per 1/24 additional score | 0.1 | 0.04 | 0.16 |  |  |  |  |  |
| Quicke 2018 | Beta coefficient (ß) | | WOMAC  function | 6 | positive ß= larger reduction function | per 1/24 additional score | 0.36 | 0.14 | 0.58 |  |  |  |  |  |

† Lee2018b is a multinomial trajectory-based study which measured weekly WOMAC pain and function over 12 weeks. 4 trajectories were identified for pain and function.

1. **Prognostic Factor Comorbidity**

| **Study** | **Effect Measure**  ***unadjusted** | **Outcome Measure** | **Follow up duration (months)** | **Outcome reference**  **(Original study)** | **Comorbidity measure** | **Comorbidity Target group**  **(Original study)** | **Effect size (Original study)** | **Low**  **95 % CI** | **High 95 % CI** | **Outcome Reference (rescaled)** | **Comorbidity Target Group (rescaled)** | **Effect size (rescaled)** | **Low 95 %CI** | **High**  **95 % CI** |
| --- | --- | --- | --- | --- | --- | --- | --- | --- | --- | --- | --- | --- | --- | --- |
| Lee2018b | OR | WOMAC  pain | 3 | Higher pain, delayed improvement (v lower pain, early improvement | Self-report comorbidity (heart disease) | presence of heart disease= 1  No heart disease =0 | 1.23 | 0.12 | 12.1 | Responder=1 | presence of heart disease= 1  No heart disease =0 | 0.81 | 0.08 | 8.33 |
| Lee2018b | OR | WOMAC  pain | 3 | Higher pain no improvement (v lower pain, early improvement) | Self-report comorbidity (HT) | presence of HT= 1  No HT =0 | 0.63 | 0.19 | 2.08 | Responder=1 | presence of HT= 1  No HT =0 | 1.59 | 0.48 | 5.26 |
| Lee2018b | OR | WOMAC  pain | 3 | Higher pain no improvement (v lower pain, early improvement) | Self-report comorbidity (diabetes) | presence of diabetes= 1  No diabetes =0 | 1.09 | 0.26 | 4.63 | Responder=1 | presence of diabetes= 1  No diabetes =0 | 0.92 | 0.22 | 3.85 |
| Lee2018b | OR | WOMAC  function | 3 | Lower function, delayed improvement (v higher function, early improvement) | Self-report comorbidity (heart disease) | presence of heart disease= 1  No heart disease =0 | 2.34 | 0.38 | 14.6 | Responder=1 | presence of heart disease= 1  No heart disease =0 | 0.43 | 0.07 | 2.63 |
| Lee2018b | OR | WOMAC  function | 3 | Lower function, delayed improvement (v higher function, early improvement) | Self-report comorbidity (HT) | presence of HT= 1  No HT =0 | 1.18 | 0.46 | 3.03 | Responder=1 | presence of HT= 1  No HT =0 | 0.85 | 0.33 | 2.17 |
| Lee2018b | OR | WOMAC  function | 3 | Lower function, delayed improvement (v higher function, early improvement) | Self-report comorbidity (diabetes) | presence of diabetes= 1  No diabetes =0 | 1.02 | 0.28 | 3.7 | Responder=1 | presence of diabetes= 1  No diabetes =0 | 0.98 | 0.27 | 3.57 |
| OLeary 2020 | OR * | GROC | 6 | Non-responder=1 | Self-report comorbidity number /18 | per 1/18 additional reported | 1.03 | 0.89 | 1.2 | Responder=1 | per 1/18 additional reported | 0.97 | 0.83 | 1.12 |
| Eyles 2016 | OR * | WOMAC-G | 6 | Non-responder=1 | Modified SCQ | Moderate (0-3) = 1  Low (0-2) =0 | 1.3 | 0.63 | 2.88 | Responder=1 | Moderate (0-3) = 1  Low (0-2) =0 | 0.77 | 0.35 | 1.59 |
| Eyles 2016 | OR * | WOMAC-G | 6 | Non-responder=1 | Modified SCQ | High (3-5) = 1  Low (0-2) =0 | 0.5 | 0.17 | 1.73 | Responder=1 | High (3-5) = 1  Low (0-2) =0 | 2 | 0.58 | 5.88 |
| Eyles 2016 | OR * | Transition scale | 6 | Non-responder=1 | Modified SCQ | Moderate (0-3) = 1  Low (0-2) =0 | 0.9 | 0.43 | 1.1 | Responder=1 | Moderate (0-3) = 1  Low (0-2) =0 | 1.11 | 0.91 | 2.33 |
| Eyles 2016 | OR * | Transition scale | 6 | Non-responder=1 | Modified SCQ | High (3-5) = 1 Low (0-2) =0 | 0.6 | 0.21 | 1.68 | Responder=1 | High (3-5) = 1  Low (0-2) =0 | 1.67 | 0.6 | 4.76 |
| Eyles 2016 | OR * | WOMAC-G + transition | 6 | Non-responder=1 | Modified SCQ | Moderate (0-3) = 1  Low (0-2) =0 | 1.1 | 0.6 | 2.05 | Responder=1 | Moderate (0-3) = 1  Low (0-2) =0 | 0.91 | 0.49 | 1.67 |
| Eyles 2016 | OR * | WOMAC-G + transition | 6 | Non-responder=1 | Modified SCQ | High (3-5) = 1 Low (0-2) =0 | 0.6 | 0.26 | 1.41 | Responder=1 | High (3-5) = 1 Low (0-2) =0 | 1.67 | 0.71 | 3.85 |
| Eyles 2014 | OR * | WOMAC-G | 6 | Responder=1 | Modified SCQ | High (3-5) =1 Low (0-2) =0 | 0.8 | 0.47 | 1.37 | Responder=1 | High (3-5) =1 Low (0-2) =0 | 0.8 | 0.47 | 1.37 |
| Eyles 2014 | OR * | WOMAC-G | 6 | Responder=1 | Modified SCQ | Very High (≥6) =1  Low (0-2) =0 | 2.2 | 0.99 | 4.95 | Responder=1 | Very High (≥6) =1  Low (0-2) =0 | 2.2 | 0.99 | 4.95 |
| Weigl 2006 | OR | WOMAC-G | 6 | Responder=1 | SCQ | Low comorbidity (≤) =1  >1 comorbidity =0 | 1.51 | 0.8 | 2.87 | Responder=1 | Higher (>1) =1  Low (≤1) = 0 | 0.66 | 0.35 | 1.25 |
| Weigl 2006 | OR | Transition scale | 6 | Responder=1 | SCQ | Low comorbidity (≤) =1  >1 comorbidity =0 | 2.75 | 1.37 | 5.5 | Responder=1 | Higher (>1) =1  Low (≤1) = 0 | 0.36 | 0.18 | 0.73 |
| Weigl 2006 | OR | Transition + WOMAC-G | 6 | Responder=1 | SCQ | Low comorbidity (≤) =1  >1 comorbidity =0 | 3.64 | 1.57 | 8.43 | Responder=1 | Higher (>1) =1  Low (≤1) = 0 | 0.27 | 0.12 | 0.64 |
| OLeary 2018a | OR | OMERACT-OARSI | 0 | Non-responder=1 | SCQ | per 1/42 additional SCQ score | 1.28 | 1.04 | 1.57 | Responder=1 | per 1/42 additional SCQ score | 0.78 | 0.64 | 0.96 |
| OLeary 2018a | OR | OMERACT-OARSI | 6 | Non-responder=1 | SCQ | per 1/42 additional SCQ score | 1.05 | 0.99 | 1.11 | Responder=1 | per 1/42 additional SCQ score | 0.95 | 0.9 | 1.01 |
| OLeary 2018a | OR | OMERACT-OARSI | 0 | Non-responder=1 | SCQ | per 1/42 additional SCQ score | 1.25 | 1.01 | 1.54 | Responder=1 | per 1/42 additional SCQ score | 0.8 | 0.65 | 0.99 |
| Ernstgard 2017 1 | OR | PA minimum | 12 | Non-responder=1 | Charnley classification | Charnley B =1  Charnley C=0 | 1 | 0.9 | 1.11 | Responder=1 | Charnley B =1  Charnley C=0 | 1 | 0.9 | 1.11 |
| Ernstgard 2017 | OR | PA minimum | 12 | Non-responder=1 | Charnley classification | Charnley C =1  Charnley A =0 | 1.15 | 1.04 | 1.28 | Responder=1 | Charnley C =1  Charnley A=0 | 0.87 | 0.78 | 0.96 |
| Dell’lsola 2020 | Beta coefficient (ß) | NRS | 3 | positive ß= larger increase pain | Charnley | Charnley B =1  Charnley A =0 | 0.38 | 0.3 | 0.46 |  |  |  |  |  |
| Dell’lsola 2020 | Beta coefficient (ß) | NRS | 3 | positive ß= larger increase pain | Charnley | Charnley C =1  Charnley A =0 | 0.49 | 0.41 | 0.56 |  |  |  |  |  |
| Dell’lsola 2020 | Beta coefficient (ß) | NRS | 12 | positive ß= larger increase pain | Charnley | Charnley B =1  Charnley A =0 | 0.5 | 0.34 | 0.67 |  |  |  |  |  |
| Dell’lsola 2020 | Beta coefficient (ß) | NRS | 12 | positive ß= larger increase pain | Charnley | Charnley C =1  Charnley A =0 | 0.71 | 0.56 | 0.86 |  |  |  |  |  |
| Nelligan 2021 | Beta coefficient (ß) | WOMAC function | 6 | positive ß= improvement in WOMAC function | Self-reported comorbidity number/13 | Per additional comorbidities reported (0-4) | 0.49 | -1.68 | 2.41 |  |  |  |  |  |
| Nelligan 2021 | Beta coefficient (ß) | NRS | 6 | positive ß= improvement in NRS pain | Self-reported comorbidity number/13 | Per additional comorbidities reported (0-4) | 0.23 | -0.21 | 0.68 |  |  |  |  |  |
| Pihl 2021 | MD | VAS | 3 | Negative less improvement (v no comorbidities) | Self-report comorbidity | 1 comorbidity=1  No comorbidities =0 | 0.2 | -0.5 | 0 |  |  |  |  |  |
| Pihl 2021 | MD | VAS | 3 | Negative less improvement (v no comorbidities | Self-report comorbidity | 2 comorbidities=1 No comorbidities =0 | 1.4 | 0.4 | 2.3 |  |  |  |  |  |
| Pihl 2021 | MD | VAS | 3 | Negative less improvement (v no comorbidities | Self-report comorbidity | 3 comorbidities=1 | 0.5 | -1.1 | 2 |  |  |  |  |  |
| Pihl 2021 | MD | VAS | 3 | Negative less improvement (v no comorbidities) | Self-report comorbidity | ≥4 comorbidity=1  No comorbidities =0 | 2.6 | -0.2 | 5.5 |  |  |  |  |  |
| Pihl 2021 | MD | 40m walk | 3 | Negative less improvement (v no comorbidities) | Self-report comorbidity | 1 comorbidity=1  No comorbidities =0 | 0.00 | -0.01 | 0.01 |  |  |  |  |  |
| Pihl 2021 | MD | 40m walk | 3 | Negative less improvement (v no comorbidities) | Self-report comorbidity | 2 comorbidities=1  No comorbidities =0 | 0.01 | -0.01 | 0 |  |  |  |  |  |
| Pihl 2021 | MD | 40m walk | 3 | Negative less improvement (v no comorbidities) | Self-report comorbidity | 3 comorbidities=1  No comorbidities =0 | 0.00 | -0.02 | 0.01 |  |  |  |  |  |
| Pihl 2021 | MD | 40m walk | 3 | Negative less improvement (v no comorbidities) | Self-report comorbidity | ≥4 comorbidity=1  No comorbidities =0 | 0.00 | 0.01 | 0.03 |  |  |  |  |  |
| Legha 2020 | MD | WOMAC  pain | 6 | Positive= less improvement in pain (v reference group | Self-report comorbidity | 3+ comorbidities= 1  Comorbidities (0-2) =0 | 0.31 | -0.17 | 0.79 |  |  |  |  |  |
| Legha 2020 | MD | WOMAC  function | 6 | Positive= less improvement in pain (v reference group | Self-report comorbidity | 3+ comorbidities= 1  Comorbidities (0-2) =0 | 0.87 | -0.74 | 2.48 |  |  |  |  |  |
| Lee 2018a | HR * | WOMAC  pain | Up to 3 | Responder=1 | Self-report comorbidity (heart) | presence of heart disease= 1  No heart disease =0 | 0.66 | 0.31 | 1.41 |  |  |  |  |  |
| Lee 2018a | HR * | WOMAC  pain | Up to 3 | Responder=1 | Self-report comorbidity (HT) | presence of HT= 1  No HT =0 | 1.14 | 0.8 | 1.63 |  |  |  |  |  |
| Lee 2018a | HR * | WOMAC  pain | Up to 3 | Responder=1 | Self-report comorbidity (diabetes) | presence of diabetes= 1  No diabetes =0 | 1.01 | 0.62 | 1.65 |  |  |  |  |  |
| Lee 2018a | HR * | WOMAC  function | Up to 3 | Responder=1 | Self-report comorbidity (heart) | presence of heart disease= 1  No heart disease =0 | 0.93 | 0.45 | 1.9 |  |  |  |  |  |
| Lee 2018a | HR * | WOMAC  function | Up to 3 | Responder=1 | Self-report comorbidity (HT) | presence of HT= 1  No HT =0 | 0.88 | 0.61 | 1.27 |  |  |  |  |  |
| Lee 2018a | HR * | WOMAC  function | Up to 3 | Responder=1 | Self-report comorbidity (diabetes) | presence of diabetes= 1  No diabetes =0 | 1.04 | 0.63 | 1.72 |  |  |  |  |  |

| **Secondary Outcome Studies examining baseline patient characteristics associated with change in willingness to undertake surgery (or undertake knee joint replacement)** | | | | | | | |
| --- | --- | --- | --- | --- | --- | --- | --- |
| **Study** | **Outcome Measure** | **Factors examined at baseline** | **Effect measure** | **Factor of interest** | **Effect Size** | **Low 95 % CI** | **High 95 % CI** |
| Teoh 2017 | Change in willingness to undertake surgery  5-point Likert scale (willing to unwilling)  Dichotomised outcome willing/not willing | Demographics, age, sex, BMI, signal joint  KOOS/HOOS  Comorbidities (number)  Depression (DASS21)  6MWT | OR (unadjusted) | Sex (female reference) | 1.10 | 0.59 | 2.05 |
|  |  |  |  | BMI (reference BMI, 30) | 0.80 | 0.44 | 1.45 |
|  |  |  |  | Depression (< 10 DASS21) | 1.15 | 0.63 | 2.12 |
|  |  |  |  | Comorbidities (number per one unit increase) | 1.02 | 0.85 | 1.23 |
|  |  |  |  | Age * adjusted OR * | 0.94 | 0.90 | 0.98 |
| Dell’lsola 2021 | Change in willingness to undertake surgery (dichotomised yes/no question) | Age, sex, BMI, demographics, comorbidities (number), physical activity, Charnley classification, fear of movement, walking difficulties, surgery, medication, previous radiographs | OR (Adjusted) | N/A | N/A | N/A | N/A |
| Gustaffsson 2022 | Time to arthroplasty at one and five years (knee joint analysed separately) | Age, sex, BMI, demographics, comorbidities (number), physical activity, QOL, Charnley classification, fear of movement, pain frequency and intensity, walking difficulties, surgery, medication | HR (Adjusted) | Age (continuous) | 1.03 | 1.02 | 1.03 |
|  |  |  |  | Sex (female reference) | 1.02 | 0.96 | 1.09 |
|  |  |  |  | BMI (overweight and obese compared to normal) | Overweight 1.24  Obese 1.25 | 1.15  1.15 | 1.35  1.37 |
|  |  |  |  | Comorbidities (1-3, ≥4 compared to 0) | 1-3 comorbid= 1.08  ≥4 = 0.98 | 0.98  0.89 | 1.18  1.09 |
|  |  |  |  | Charnley classification (reference A) | Charnley B= 1.34  Charnley C= 1.04 | 1.24  0.97 | 1.44  1.12 |
| Gwynne-Jones 2020 | Undertook TJR surgery (Yes/No) | Age, sex, BMI, affected Joint  KL grade  Oxford hip/knee (OHKS)  SF-12 | HR (Adjusted) | Age (continuous) | 1.14 | 0.97 | 1.33 |
|  |  |  |  | Sex (female reference) | 0.69 | 0.42 | 1.11 |
|  |  |  |  | KL grade (reference KL 1-2) | KL grade 3 2.52  KL Grade 4 8.27 | 1.29  3.91 | 4.95  17.48 |

1. ^2^ Original data from Gwynne-Jones 2018 rescaled from age per 5 years to age per one year [↑](#footnote-ref-1)
2. [↑](#footnote-ref-2)
