# Supplementary material for: Evidence for key individual characteristics associated with outcomes following combined first-line interventions for knee osteoarthritis: A systematic review
Source: PLoS One. 2023 Apr 11;18(4):e0284249. doi: 10.1371/journal.pone.0284249 (PMC10089365; doi:10.1371/journal.pone.0284249)
Supplement: S5 Table — (DOCX) [file pone.0284249.s006.docx]

| Table 3 Rating of certainty and plain text interpretation of results | | | | |
| --- | --- | --- | --- | --- |
| **Prognostic Factor** | **Effect Measure** | **GRADE interpretation** | **Certainty in evidence** | **Plain text interpretation** |
| Age | OR | Precise 5/8 studies  Little variability point estimate (0.9-1) most studies  Inconsistency in one study measuring PA threshold  Moderate ROB 7/8 studies  3/7 unadjusted effect estimate | Moderate | Small negative effect of increasing age on the probability of positive response (less than 10% reduction in odds) |
| Sex  (female v male) | OR | Positive effect measure 7/8 studies (OR between 1-3)  Imprecise 7/8 studies  ROB moderate all 8 studies with 6/8 in 4 or more QUIPS domains | Low  Inconsistency and risk of bias | Females (compared to males) have an increased probability of having a positive response (up to 2-3 times the odds) |
| BMI | OR | 5/5 moderate ROB – 3/5 unadjusted effect estimate  Consistent effect measure 3/5 studies (OR 0.93-1)  Inconsistency in one study (multidirectional)  Larger negative effect measure in the study dichotomised BMI | Very low  Risk of bias and limited number of studies | Small negative effect of increasing BMI on the probability of positive response (less than 10% reduction in odds) |
| Depression | OR | 5/7 moderate ROB – 3/7 unadjusted effect estimate  4/7 studies small negative effect measure (OR 0.9-1)  2 studies positive effect measure (OR 1.2 and 3.3)  Inconsistency within 1 study(multidirectional) | Very low  Inconsistency, risk of bias, and imprecision | Insufficient evidence to reliably determine the direction or magnitude of the relationship between depression and outcome |
| Comorbidity | OR | Moderate ROB 6/7 studies  Inconsistent (multidirectional)effect measure  Imprecise effect measures | Very low  Imprecision, inconsistency, and risk of bias | Insufficient evidence to reliably determine the direction or magnitude of the relationship between comorbidity and outcome |
| Baseline imaging | OR | Low to moderate ROB (4 studies reported baseline imaging) Indirectness high due to variation in imaging modalities, different cut-off points and different outcome measures for responder analysis.  Imprecise effect measures for all studies | Low  Indirectness  Imprecise | More severe osteoarthritis severity was associated with a reduced positive response- evidence is uncertain due to limited studies, imprecision and large variation in study methods |
| The outcome of interest was a change in pain and function for which a single pooled estimate was not calculated, and only a narrative synthesis was performed. The rating of certainty was based on recommendations for the use of GRADE for the assessment of prognostic factors and rating certainty in the absence of a single effect estimate ^28 29^. Indirectness was considered relevant for the certainty of all effect estimates due to variation in intervention components and a large variety of outcome measures and follow-up duration. The effect estimates and 95 % CI for all (OR, MD, RR, HR, beta coefficient) are attached in Appendix 3. | | | | |
