# Supplementary material for: Evidence for key individual characteristics associated with outcomes following combined first-line interventions for knee osteoarthritis: A systematic review
Source: PLoS One. 2023 Apr 11;18(4):e0284249. doi: 10.1371/journal.pone.0284249 (PMC10089365; doi:10.1371/journal.pone.0284249)
Supplement: S1 File — (PDF) [file pone.0284249.s007.pdf]

Systematic review

To edit the record click *Start an update* below. This will create a new version of the record - the existing version will remain unchanged.

1. * Review title.

Give the title of the review in English

Patient characteristics that predict the outcome following a multicomponent non-surgical intervention for knee osteoarthritis: a systematic review and meta-analysis

2. Original language title.

For reviews in languages other than English, give the title in the original language. This will be displayed with the English language title.

3. * Anticipated or actual start date.

Give the date the systematic review started or is expected to start.

12/05/2021

4. * Anticipated completion date.

Give the date by which the review is expected to be completed.

20/12/2021

5. * Stage of review at time of this submission.

Tick the boxes to show which review tasks have been started and which have been completed.
Update this field each time any amendments are made to a published record.

Reviews that have started data extraction (at the time of initial submission) are not eligible for inclusion in PROSPERO.
If there is later evidence that incorrect status and/or completion date has been supplied, the published PROSPERO record will be marked as retracted.

This field uses answers to initial screening questions. It cannot be edited until after registration.

The review has not yet started: No

Review stage	Started	Completed
Preliminary searches	Yes	No
Piloting of the study selection process	Yes	No
Formal screening of search results against eligibility criteria	No	No
Data extraction	No	No
Risk of bias (quality) assessment	No	No
Data analysis	No	No

Provide any other relevant information about the stage of the review here.

6. * Named contact.

The named contact is the guarantor for the accuracy of the information in the register record. This may be any member of the review team.

Jacqui Couldrick

Email salutation (e.g. "Dr Smith" or "Joanne") for correspondence:
Jacqui

7. * Named contact email.

Give the electronic email address of the named contact.

jacqui.couldrick@canberra.edu.au

8. Named contact address

PLEASE NOTE this information will be published in the PROSPERO record so please do not enter private information, i.e. personal home address

Give the full institutional/organisational postal address for the named contact.

Faculty of Health University of Canberra 11 Kirinari St Bruce ACT 2617

9. Named contact phone number.

Give the telephone number for the named contact, including international dialling code.

+61 418270358

10. * Organisational affiliation of the review.

Full title of the organisational affiliations for this review and website address if available. This field may be completed as 'None' if the review is not affiliated to any organisation.

University of Canberra

Organisation web address:
http://www.canberra.edu.au/about-uc/faculties/health

11. * Review team members and their organisational affiliations.

Give the personal details and the organisational affiliations of each member of the review team. Affiliation refers to groups or organisations to which review team members belong.
NOTE: email and country now MUST be entered for each person, unless you are amending a published record.

Ms Jacqui Couldrick. Faculty of Health University of Canberra
Professor Jennie Scarvell. Faculty of Health University of Canberra
Professor Diana Perriman. Trauma and Orthopaedic Research Unit, The Canberra Hospital . College of Medicine and Health Sciences, the Australian National University.
Mrs Denika Silva. Faculty of Health University of Canberra
Assistant/Associate Professor Theo Niyonsenga. University of Canberra

12. * Funding sources/sponsors.

Details of the individuals, organizations, groups, companies or other legal entities who have funded or sponsored the review.

None

Grant number(s)
State the funder, grant or award number and the date of award

None

13. * Conflicts of interest.

List actual or perceived conflicts of interest (financial or academic).

None

14. Collaborators.

Give the name and affiliation of any individuals or organisations who are working on the review but who are not listed as review team members. **NOTE: email and country must be completed for each person, unless you are amending a published record.**

15. * Review question.

State the review question(s) clearly and precisely. It may be appropriate to break very broad questions down into a series of related more specific questions. Questions may be framed or refined using PI(E)COS or similar where relevant.

The aim of this study is to identify patient characteristics (prognostic factors at baseline) that predict the outcome (change in pain or function) following a multicomponent non-surgical intervention for knee osteoarthritis

16. * Searches.

State the sources that will be searched (e.g. Medline). Give the search dates, and any restrictions (e.g. language or publication date). Do NOT enter the full search strategy (it may be provided as a link or attachment below.)

MEDLINE
CINAHL
Scopus
Web of Science Core Collection
Cochrane Library

No restriction for language or publication date.
Electronic searches will be complemented by manual searching of reference lists from included studies and previous reviews in addition to clinical practice guidelines for knee osteoarthritis.

17. URL to search strategy.

Upload a file with your search strategy, or an example of a search strategy for a specific database, (including the keywords) in pdf or word format. In doing so you are consenting to the file being made publicly accessible.

Or provide a URL or link to the strategy. Do NOT provide links to your search **results**.

https://www.crd.york.ac.uk/PROSPEROFILES/234398_STRATEGY_20210508.pdf

Yes I give permission for this file to be made publicly available

18. * Condition or domain being studied.

Give a short description of the disease, condition or healthcare domain being studied in your systematic review.

Those with knee osteoarthritis that have undertaken a multicomponent first line intervention and who have not undergone joint replacement surgery will be the focus of this review. Current international clinical practice guidelines (CPG) recommend that all those with knee osteoarthritis trial the core evidence based non-pharmacological interventions consisting of land-based exercise, education or self-management and weight management prior to considering TKR surgery (Bannaru et al, 2019).

<u>Reference</u>

Bannuru et al (2019). "OARSI guidelines for the non-surgical management of knee, hip, and polyarticular osteoarthritis." Osteoarthritis and Cartilage 27(11): 1578-1589.

19. * Participants/population.

Specify the participants or populations being studied in the review. The preferred format includes details of both inclusion and exclusion criteria.

Inclusion Criteria:

Adults (over 18 years) diagnosed with knee osteoarthritis using clinical or radiological methods:

1. American College of Rheumatology Criteria (ACR) (Altman et al, 1986)
2. NICE Guidelines (2014)
3. Kellgren-Lawrence classification (Kohn et al, 2016).

Knee OA may be diagnosed by Xray or MRI

Knee OA may be diagnosed clinically by a healthcare professional

Exclusion Criteria:

Participants with rheumatoid arthritis or other defined inflammatory rheumatological problems

Participants with pain not attributable to knee OA

Post-operative patients (following joint replacement or other surgical procedures for the knee)

20. * Intervention(s), exposure(s).

Give full and clear descriptions or definitions of the interventions or the exposures to be reviewed. The preferred format includes details of both inclusion and exclusion criteria.

Inclusion Criteria

The intervention must include a multicomponent non-surgical intervention (Bannaru et al, 2019) consisting of:

1. Structured land-based exercise program (including strengthening, aerobic, balance or neuromuscular exercise or mind body exercise such as tai chi or yoga).

AND at least one of the following components

2. Arthritis education or self-management
3. Dietary management/weight loss

The intervention may include other nonpharmacological interventions (such as massage therapy or orthotics or other multidisciplinary interventions).

Exclusion Criteria:

1. Intervention not consisting of at least 2 components (Exercise and/or education or weight management).
2. Pharmacological interventions such as joint injections or medications.

Reference:

Bannuru, R. R., et al. (2019). "OARSI guidelines for the non-surgical management of knee, hip, and polyarticular osteoarthritis." Osteoarthritis and Cartilage 27(11): 1578-1589.

21. * Comparator(s)/control.

Where relevant, give details of the alternatives against which the intervention/exposure will be compared (e.g. another intervention or a non-exposed control group). The preferred format includes details of both inclusion and exclusion criteria.

None

22. * Types of study to be included.

Give details of the study designs (e.g. RCT) that are eligible for inclusion in the review. The preferred format includes both inclusion and exclusion criteria. If there are no restrictions on the types of study, this should be stated.

Inclusion criteria:

Not restricted to any study design and may include data from RCTs and registries

The study must identify prognostic factors at baseline and report a statistical association with the outcome.

As a minimum, the baseline prognostic factors of interest will include demographic information (sex and age). Other prognostic factors of interest are BMI, number of comorbidities, psychological characteristics, and baseline OA severity (pain or KL grade).

Exclusion Criteria:

Studies not evaluating a relationship between a baseline variable (predictor) and an outcome.

23. Context.

Give summary details of the setting or other relevant characteristics, which help define the inclusion or exclusion criteria.

Several non-surgical and non-pharmacological interventions exist for the management of hip and knee OA that include OA education, structured land-based exercise programs and dietary management. Examples of these programs include the Good Living Arthritis Denmark program (GLA:D) and the Better Management of OA (BOA) program which both consist of 2 education sessions and 12 physiotherapy led exercise sessions conducted in either a group setting or delivered as supervised home exercise program. These programs may be run in community-based settings, hospitals or a private setting and more recently via telehealth. Non-surgical multidisciplinary programs occur in a hospital setting that are physiotherapy led and include OA education/self-management and structured exercise programs in addition to other multidisciplinary care such as occupational therapy and dietetics.

Outcomes following these programs have demonstrated improvements in pain and function and there is evidence that supervised non-surgical treatment can delay the likelihood of a total knee replacement (TKR) up to 2 years in 68 % of patients (Skou et al 2018).

There is recent interest in determining who may benefit from these programs or which patient characteristics may predict the outcome following these first-line interventions. Understanding the predictors of a response to a non-operative treatment program may assist in individualising a person's care and determining who is suitable for non-operative care. Prognostic factor research aims to identify factors that are associated with a particular clinical outcome. This systematic review will investigate the baseline characteristics or predictors of a response to a multicomponent non-surgical intervention for knee OA.

24. * Main outcome(s).

Give the pre-specified main (most important) outcomes of the review, including details of how the outcome is defined and measured and when these measurement are made, if these are part of the review inclusion criteria.

Change in pain or function from baseline to follow up measured with any patient reported outcomes such as: WOMAC, KOOS, Oxford knee scale or self-reported pain scales such as numerical pain rating scale (NPRS) and visual analogue scale (VAS).

Improvement in knee pain and function can be dichotomised to include a responder or non-responder based on a predetermined value in change in pain such as a minimal clinically important difference (MCID) or minimum percentage change.

Measures of effect

Measures of association such as odds ratio (OR) or risk ratio (RR) and confidence intervals or standard error and regression coefficient and confidence intervals.

25. * Additional outcome(s).

List the pre-specified additional outcomes of the review, with a similar level of detail to that required for main outcomes. Where there are no additional outcomes please state ‘None’ or ‘Not applicable’ as appropriate to the review

Change in willingness to undertake surgery or undertake TKR

Measures of effect

Measures of association such as odds ratio (OR) or risk ratio (RR) and confidence intervals or standard error and regression coefficient and confidence intervals.

26. * Data extraction (selection and coding).

Describe how studies will be selected for inclusion. State what data will be extracted or obtained. State how this will be done and recorded.

Covidence software will be used to manage the review process between reviewers at the University of Canberra. Titles, abstracts, and full text of studies retrieved using the search strategy will be screened independently by two reviewers to identify studies that potentially meet the inclusion criteria outlined above. Consensus will be used to resolve any disagreements and a third reviewer will be consulted.

A modified version of the Charms-PF checklist of key items to be extracted from the study will be used (Riley et al, 2019).

<u>Extracted data will include:</u>

Aim of the study

Type of study and source of data
Sample size and missing data
Inclusion/exclusion criteria
Description of Intervention
Outcomes to be predicted and the time frame for prediction
Number and type of prognostic factors included in analysis
Type of analysis used and description of methods of modelling and inclusion/exclusion of prognostic factors
Adjusted or unadjusted factors used
Reporting of effect estimate and confidence interval for each prognostic factor reported

Reference:
Riley, R. D., et al. (2019). "A guide to systematic review and meta-analysis of prognostic factor studies." BMJ 364: k4597.

27. * Risk of bias (quality) assessment.

State which characteristics of the studies will be assessed and/or any formal risk of bias/quality assessment tools that will be used.

The Charms-PF checklist of key items to be extracted from the study will be used to critically appraise and extract data.

The QUIPS tool (quality in prognostic factor studies) will be used to examine each study's risk of bias (Riley et al, 2019). The QUIPS tool is recommended for evaluation of prognostic factor studies and consists of 6 domains - study participation (selection bias), study attrition, prognostic factor measurement (misclassification bias), outcome measurement (detection bias), study confounders and statistical analysis and reporting. Each domain will be assessed for overall quality by two independent reviewers. Each domain will be rated as low, moderate, or high risk of bias and consensus will be made by the 2 assessors.

Reference:
Riley et al. A guide to systematic review and meta-analysis of prognostic factor studies BMJ 2019; 364 :k4597

28. * Strategy for data synthesis.

Describe the methods you plan to use to synthesise data. This **must not be generic text** but should be **specific to your review** and describe how the proposed approach will be applied to your data.
If meta-analysis is planned, describe the models to be used, methods to explore statistical heterogeneity, and software package to be used.

A narrative synthesis of the findings from the included studies will be performed which will include the intervention components, the statistical methods used, outcome measures and the predictor variables.
The results of the findings from each included study will be presented in a table with the effect estimate presented for each predictor variable, for example odds ratio (OR) with corresponding standard error or confidence interval. Where possible, reporting of both adjusted and unadjusted prognostic factors will occur.
A meta-analysis will be performed using either random effects or fixed effect model. A minimum of 3 studies per prognostic factor of interest will be required to perform a metanalysis. It is anticipated there will be limited scope for meta-analysis because of the range of different types of interventions, outcomes measured, and the high number of prognostic factors assessed across the small number of existing trials.

29. * Analysis of subgroups or subsets.

State any planned investigation of 'subgroups'. Be clear and specific about which type of study or participant will be included in each group or covariate investigated. State the planned analytic approach.

Subgroup analysis may be conducted for:
1. Different settings or interventions – such as hospital based or primary care programs (multicomponent and multidisciplinary programs).
2. Affected joint- knee data will be analysed separately where possible, but a subgroup analysis may occur for data where hip/knee OA is presented together.

30. * Type and method of review.

Select the type of review, review method and health area from the lists below.

Type of review

Cost effectiveness	No
Diagnostic	No
Epidemiologic	No
Individual patient data (IPD) meta-analysis	No
Intervention	No
Living systematic review	No
Meta-analysis	Yes
Methodology	No
Narrative synthesis	Yes
Network meta-analysis	No
Pre-clinical	No
Prevention	No
Prognostic	Yes
Prospective meta-analysis (PMA)	No
Review of reviews	No
Service delivery	No
Synthesis of qualitative studies	No
Systematic review	Yes
Other	No

Health area of the review

Alcohol/substance misuse/abuse	No
Blood and immune system	No
Cancer	No
Cardiovascular	No
Care of the elderly	No
Child health	No
Complementary therapies	No
COVID-19	No
Crime and justice	No

Dental	No
Digestive system	No
Ear, nose and throat	No
Education	No
Endocrine and metabolic disorders	No
Eye disorders	No
General interest	No
Genetics	No
Health inequalities/health equity	No
Infections and infestations	No
International development	No
Mental health and behavioural conditions	No
Musculoskeletal	Yes
Neurological	No
Nursing	No
Obstetrics and gynaecology	No
Oral health	No
Palliative care	No
Perioperative care	No
Physiotherapy	Yes
Pregnancy and childbirth	No
Public health (including social determinants of health)	No
Rehabilitation	Yes
Respiratory disorders	No
Service delivery	No
Skin disorders	No
Social care	No
Surgery	No
Tropical Medicine	No

Urological	No
Wounds, injuries and accidents	No
Violence and abuse	No

31. Language.

Select each language individually to add it to the list below, use the bin icon to remove any added in error.

English

There is an English language summary.

32. * Country.

Select the country in which the review is being carried out. For multi-national collaborations select all the countries involved.

Australia

33. Other registration details.

Name any other organisation where the systematic review title or protocol is registered (e.g. Campbell, or The Joanna Briggs Institute) together with any unique identification number assigned by them.
If extracted data will be stored and made available through a repository such as the Systematic Review Data Repository (SRDR), details and a link should be included here. If none, leave blank.

34. Reference and/or URL for published protocol.

If the protocol for this review is published provide details (authors, title and journal details, preferably in Vancouver format)

Yes I give permission for this file to be made publicly available

35. Dissemination plans.

Do you intend to publish the review on completion?

Yes

Publication in peer-reviewed journal
Presentation at relevant conference such as physiotherapy or musculoskeletal conference

36. Keywords.

Give words or phrases that best describe the review. Separate keywords with a semicolon or new line. Keywords help PROSPERO users find your review (keywords do not appear in the public record but are included in searches). Be as specific and precise as possible. Avoid acronyms and abbreviations unless these are in wide use.

Predictive factors
knee osteoarthritis
Multicomponent interventions
Non-surgical interventions

37. Details of any existing review of the same topic by the same authors.

If you are registering an update of an existing review give details of the earlier versions and include a full bibliographic reference, if available.

None

38. * Current review status.

Update review status when the review is completed and when it is published.
New registrations must be ongoing so this field is not editable for initial submission.

Review_Ongoing

39. Any additional information.

Provide any other information relevant to the registration of this review.

40. Details of final report/publication(s) or preprints if available.

Leave empty until publication details are available OR you have a link to a preprint (NOTE: this field is not editable for initial submission).
List authors, title and journal details preferably in Vancouver format.
