# Supplementary material for: Evidence for key individual characteristics associated with outcomes following combined first-line interventions for knee osteoarthritis: A systematic review
Source: PLoS One. 2023 Apr 11;18(4):e0284249. doi: 10.1371/journal.pone.0284249 (PMC10089365; doi:10.1371/journal.pone.0284249)
Supplement: Attachment — Submitted filename: Response to reviewers_3Mar2023.docx [file pone.0284249.s008.docx]

**3rd March 2023**

Germain Honvo, Ph.D.
Academic Editor
PLOS ONE

### Evidence for key individual characteristics associated with outcomes following combined first-line interventions for knee osteoarthritis: a systematic review.

**Response to reviewers [manuscript PONE-D-22-32794]**

Dear Editor

Thank you for accepting minor corrections to the manuscript

Please find below the author’s response to the reviewer’s comments

Kind regards,

Jacqui Couldrick

Corresponding author

Tel. +61418270358

[Jacqui.couldrick@canberra.edu.au](mailto:Jacqui.couldrick@canberra.edu.au)

***Reviewer #1:***

***Comment 1: The title does not match the content of the manuscript, since there are a lot of factors which are not included.***

We have amended our title to “Evidence for key individual characteristics associated with outcomes following combined first-line interventions for knee osteoarthritis: a systematic review”.

***Comment 2: It would be meaningful to indicate what the authors mean by “responders”.***

In this review, the term ‘’responder(s)” refers to whether a person has improved in either a pain or function outcome measure. Our review evaluated a change in pain or function measure from baseline to follow-up post-intervention and examined factors that may be associated with this change (or response).

The definition of the person’s “response” to the intervention was determined by each study/author(s). The response may be positive or negative and depends on what outcome measure was used to evaluate the response. Although a responder was frequently defined based on a valid clinically meaningful value such as MCID (minimum clinically important difference), this was not the case for all studies and may have been arbitrarily determined.

We have made changes to the paper and clearly stated the definition of “responder” (please refer to lines 190-196, p.9 and 271-290, p. 17-18). Most studies reported an odds ratio which makes interpretation much simpler. For continuous outcome measures, the direction and magnitude of the regression coefficient determine the extent of the positive or negative response but frequently did not reach the threshold of a minimally important difference (Dell'Isola et al., 2020; Pihl et al., 2021)

Lines 190-196 (p.9)

“The term ‘responder’ was used to refer to whether a person had improved in either a pain or function outcome measure. This response may be a positive or a negative response to the treatment and will depend on the outcome measure used to evaluate the response.

For all observations to be on a common scale, the OR and 95% CI for the prognostic factors of age, sex, BMI, comorbidity, and depression were rescaled when necessary, such that an odds ratio greater than 1 represents a positive response.”

Lines 268-283 (p.17-18)

“Table 2 briefly summarises each study’s “responder” definition. There was a range of different responder definitions including:

The OMERACT-OARSI responder criteria- a composite measure that uses improvements in pain, function and the patient’s global assessment of improvement (Pham et al., 2003).

The patient’s global assessment of improvement in pain or function (Likert scale). The scale is dichotomised to reflect a responder or non-responder cut-off value (O'Leary et al., 2020).

Patient-reported outcome measures such as WOMAC pain or function or WOMAC-global. A range of cut-off points were based on a minimally clinically important difference (MCID).

Multiple responder definitions constructed within a single study which produced several similar effect measures (Eyles et al., 2014; Eyles et al., 2016; O'Leary et al., 2018; Weigl et al., 2006).

Pain scales such as NRS pain (0-10) were dichotomised. A responder was defined as having greater than 50 % reduction in pain following the intervention (Tanaka et al., 2021)

***Comment 3: The inclusion criteria are unclear, since it is broadly stated to be prognostic factor (it is properly demographics, BMI, comorbidities, psychological factors, and baseline disease severity). Studies on e.g. baseline physical activity levels, sensitization of the nerves and inflammatory mediators have been conducted but these are not included here. Also, psychological factors are reduced to “depression” but factors such as pain catastrophizing is often found to predict treatment outcomes for patients with osteoarthritis****.*

The original intention of this systematic review was to explore prognostic factors associated with outcomes following first-line interventions. The inclusion criteria on PROSPERO (protocol number CRD42021234398 [www.crd.york.ac.uk/prospero](http://www.crd.york.ac.uk/prospero)) stated the study must: “identify a prognostic factor at baseline and report a statistical association with the outcome. As a minimum, the baseline prognostic factors of interest will include demographic information (sex and age) and other prognostic factors of interest are BMI, comorbidities, psychological characteristics, and baseline OA severity”.

As our review progressed, we felt we had to narrow the review and focus on this subset of factors. Many of the studies evaluated contained over 15-20 baseline factors. Thus, to be able to make any meaningful conclusions in this review, we chose to prioritise these factors as more studies had evaluated these.

Table 2 (p.20) lists the number and types of prognostic factors evaluated in each study. This shows the large range of factors that were identified. Other factors included psychological variables (self-efficacy, fear of movement, pain catastrophising, mindfulness), baseline physical activity level, therapeutic alliance, patient expectations of treatment and demographic factors (e.g. education level).

It is acknowledged that our focus on a subset of factors may be considered a limitation, however we felt necessary to make any useful conclusions. Past prognostic factor studies have encountered a similar problem and future research into this area should chose to narrow the focus on one or two factors (Riley et al., 2021). As evidence evolves, a future review could focus on factors such as pain sensitisation or psychological factors such as pain catastrophising.

The limitation section of this review has been amended to includes a paragraph on the limitation of a focussed review and suggestions for future research (lines 609-617, p. 36).

“…We focussed our review on key prognostic factors identified in our protocol which included demographics (age and sex), BMI, psychological factors, and OA severity (imaging). This may be considered a limitation of this review as we missed potentially important factors. Our initial research question was broad, and a more specific focus is preferable in order to make useful conclusions (Riley et al., 2021). It is acknowledged that some factors were not considered in detail. For example, several recent studies examine the association between pre-treatment pain sensitisation and outcomes following exercise therapy (Hansen et al., 2021; Hattori et al., 2022; O'Leary et al., 2018). As further evidence emerges, a systematic review focussing specifically on factors such as pain sensitisation may be warranted.”

***Comment 4: It is unclear which types of statistical analysis are “allowed” for the studies to be included in the review (e.g., is this only studies using correlation/regressions or do you allow studies that subgroup patients based on clinical outcomes/treatment outcomes?)***

Our review included studies that evaluated the association between a baseline factor (predictor variable) and the outcome. To avoid any confusion between subgroup analysis or treatment moderator studies, we specified the strength of association was to be measured by the size of the beta coefficient, odds ratio (OR), risk ratio (RR) or hazard ratio (HR) and the confidence interval (lines 122-123, p.6, lines 182-184, p.9).

We excluded treatment moderator studies or subgroup analysis where the factor measured at baseline may result in a different treatment effect size (Kraemer et al., 2006). We have updated the exclusion criteria to clarify this (lines 131-134, p.7)

“Studies that examined treatment moderators or subgroup analysis that reported treatment effect sizes for baseline prognostic factors were excluded.”

***Comment 5: The studies have a follow-up ranging from just after completing the intervention to 12-months following the intervention and the prediction of these outcomes will be very different.***

A finding of this review was the variation found across the studies over a range of dimensions, one of which includes the follow-up duration of the interventions. The included studies did have a wide distribution of follow up duration from immediately post intervention and 3-, 6- and 12-months post intervention. When visually inspecting this graph, it is unlikely the conclusions will change for this review as there are limited studies in each time point for each prognostic factor. Our supplementary file (S3_fig_followupduration_joint) was included in the submission to visually demonstrate this finding.

A similar problem was encountered when the data had been combined with both the hip and knee joint (see supplementary file (S3_fig_followupduration_joint) and was discussed as a limitation of this review (lines 604-607, p.36).

***Comment 6: it is unclear why the data in figures 3-5 are not utilized for at meta-analysis.***

A meta-analysis was deemed not appropriate due to heterogeneity. The studies varied in too many dimensions, including whether the hip and knee joint were analysed together or separately, the measurement of prognostic factors at baseline, intervention components, follow-up duration which ranged from immediate to 12 months, outcome measures and definition of responder, and the statistical methods. A meta-analysis would have to ignore these variations or focus on a very small subset of factors that would be unlikely to produce a meaningful estimate.

The strength and limitation section has been updated to acknowledge that we were unable to make any quantitative comparisons between the studies and did not conduct a meta-analysis (lines 600-604, p.36).

“A meta-analysis was not able to be performed due to each study varying on many dimensions such as the intervention components, the joint analysed, the follow-up duration, outcomes measures, the definition of a responder, prognostic factor measurement and the statistical methods.”

***Comment 7: please use the first section of the discussion as a summary of the study findings.***

Thank you, we have revised paragraph 1 of the discussion section with a summary of the study’s findings (lines 515 -526, p.33).

“This systematic review is the first to evaluate prognostic factors associated with pain and function outcomes following combined first-line interventions of exercise therapy, OA education or weight loss for knee OA. The rationale of this review was to identify individual characteristics that may influence a person's response to combined first-line interventions for knee OA. A meta-analysis was not able to be performed due to study heterogeneity, instead, a narrative synthesis and data visualisation was conducted. Thirty-two studies were included in this review. We found being female was associated with 2-3 times increased odds of a positive response. Older age was associated with a lower odds of responding which was unlikely to be of clinical relevance. We could not conclude whether BMI, those with comorbidities or depression and OA severity (imaging) was associated with a positive response following combined first line interventions”

***Reviewer #2: Jorge Hugo Villafane***

***Comment 1: Abstract: Conclusions: You can delete the last sentence.***

The following sentence has been deleted from the abstract.

“Future research using IPD meta-analysis may help overcome some of the challenges found in this review, allowing for standardisation of inclusion and exclusion criteria, a consistent study methodology and evaluation of larger datasets.”

***Comment 2: Introduction- suggested use of references by reviewer 2***

<https://doi.org/10.1007/s10067-018-4270-4>

<https://doi.org/10.1093/pm/pnab301>

<https://doi.org/10.1097/TGR.0000000000000328>

The authors have reviewed these articles relating to pain sensitisation in knee OA. The papers suggested by the author do not meet our inclusion criteria as they do not examine a first-line intervention of exercise, education, and weight management.

Pain sensitisation is an interesting and emerging area of research in knee osteoarthritis. We had included one study in our review that specifically examined pain sensitisation and the association with a nonresponse following a physiotherapy led exercise, education and weight loss advice for those with knee osteoarthritis (O'Leary et al., 2018) <https://doi.org/10.1097/j.pain.0000000000001288> .

Two further studies that examined pain sensitisation and outcomes following a first-line intervention were excluded from our review as they did not meet our inclusion criteria. A 2022 study examined pain sensitisation and the association with the effectiveness of exercise therapy in patients with hip and knee osteoarthritis. This was excluded from our review based on the statistical analysis and not reporting an association measure (Hattori et al., 2022) <https://doi.org/10.1155/2022/4323045>. A second study (abstract only) examined the pre-treatment pain sensitivity and the association with outcomes following exercise therapy and an interim analysis suggesting that those with higher baseline pain sensitivity may have worse outcomes <https://doi.org/10.1016/j.joca.2021.02.476> (Hansen et al., 2021).

These studies have been added to the reasons for excluded studies (line 229-233, p.11).

***Comment 3: Discussion: In the first paragraph, could you be present the result in brief.***

We have revised paragraph 1 of the discussion section and presented the results in brief (lines 511-524, p.33).

Please refer to Comment 7 (reviewer #1).

***Reviewer #3: Donald Manlapaz***

***Thank you for the opportunity to read the manuscript. The systematic review comprehensively discussed the factors such as age, sex, BMI, OA severity that are associated with the response to a first-line intervention for knee OA. The structure of the manuscript is complete and easy to read.***

Thankyou very much for your review and all comments were very helpful.

### References

1 Dell'Isola, A., Jonsson, T.et al. Factors Associated With the Outcome of a First-Line Intervention for Patients With Hip or Knee Osteoarthritis or Both: Data From the BOA Register. Phys Ther 2020; 100: 1771-1781, <https://doi.org/10.1093/ptj/pzaa113>.

2 Eyles, J. P., Lucas, B. R.et al. Does clinical presentation predict response to a nonsurgical chronic disease management program for endstage hip and knee osteoarthritis? J Rheumatol 2014; 41: 2223-2231, <https://doi.org/10.3899/jrheum.131475>.

3 Eyles, J. P., Mills, K.et al. Can We Predict Those With Osteoarthritis Who Will Worsen Following a Chronic Disease Management Program? Arthritis Care Res 2016; 68: 1268-1277, <https://doi.org/10.1002/acr.22836>.

4 Hansen, S., Bjarke Vaegter, H.et al. Pre-treatment pain sensitivity is associated with outcome following exercise therapy in patients with knee osteoarthritis - an observational study. Osteoarthritis and Cartilage 2021; 29: S367, <https://doi.org/10.1016/j.joca.2021.02.476>.

5 Hattori, T., Shimo, K.et al. Pain Sensitization and Neuropathic Pain-like Symptoms Associated with Effectiveness of Exercise Therapy in Patients with Hip and Knee Osteoarthritis. Pain Res Manag 2022; 2022: 4323045, <https://doi.org/10.1155/2022/4323045>.

6 Kraemer, H. C., Frank, E.et al. Moderators of Treatment Outcomes Clinical, Research, and Policy Importance. JAMA 2006; 296: 1286-1289, <https://doi.org/10.1001/jama.296.10.1286>.

7 O'Leary, H., Smart, K. M.et al. Pain sensitization associated with nonresponse after physiotherapy in people with knee osteoarthritis. Pain 2018; 159: 1877-1886, <https://doi.org/10.1097/j.pain.0000000000001288>.

8 O'Leary, S., Raymer, M.et al. Patient characteristics associated with a poor response to non-surgical multidisciplinary management of knee osteoarthritis: a multisite prospective longitudinal study in an advanced practice physiotherapist-led tertiary service. BMJ Open 2020; 10: e037070, <https://doi.org/10.1136/bmjopen-2020-037070>.

9 Pham, T., Van Der Heijde, D.et al. Outcome variables for osteoarthritis clinical trials: The OMERACT-OARSI set of responder criteria. J Rheumatol 2003; 30: 1648-1654, <https://www.ncbi.nlm.nih.gov/pubmed/12858473>

10 Pihl, K., Roos, E. M.et al. Associations between comorbidities and immediate and one-year outcomes following supervised exercise therapy and patient education - A cohort study of 24,513 individuals with knee or hip osteoarthritis. Osteoarthritis Cartilage 2021; 29: 39-49, <https://doi.org/10.1016/j.joca.2020.11.001>.

11 Riley, R. D., Stewart, L. A.et al. (2021). Individual Participant Data Meta‐Analysis for Healthcare Research. In *Individual Participant Data Meta‐Analysis* (pp. 1-6). <https://doi.org/10.1002/9781119333784.ch1>

12 Tanaka, S., Nishigami, T.et al. Identifying participants with knee osteoarthritis likely to benefit from physical therapy education and exercise: A hypothesis-generating study. Eur J Pain 2021; 25: 485-496, <https://doi.org/10.1002/ejp.1687>.

13 Weigl, M., Angst, F.et al. Predictors for response to rehabilitation in patients with hip or knee osteoarthritis: a comparison of logistic regression models with three different definitions of responder. Osteoarthritis Cartilage 2006; 14: 641-651, <https://doi.org/10.1016/j.joca.2006.01.001>.
